# Photocatalytic air purification mimicking the self-cleaning process of the atmosphere

**DOI:** 10.1038/s41467-021-22839-0

**Published:** 2021-05-05

**Authors:** Fei He, Woojung Jeon, Wonyong Choi

**Affiliations:** Division of Environmental Science and Engineering & Department of Chemical Engineering, Pohang University of Science and Technology (POSTECH), Pohang, Korea

**Keywords:** Pollution remediation, Photocatalysis, Pollution remediation

## Abstract

Photocatalytic air purification is a promising technology that mimics nature’s photochemical process, but its practical applications are still limited despite considerable research efforts in recent decades. Here, we briefly discuss the progress and challenges associated with this technology.

## Photocatalysis as an eco-friendly method of air cleaning

Air pollution severely endangers human health and the environment and requires highly efficient and viable treatment technologies^1^. There are various technologies for controlling air pollutants, among which adsorption using activated carbons or highly porous materials is the most commonly practised method. However, the adsorbents need to be frequently replaced, the adsorption efficiency is significantly decreased under humid conditions due to the competing adsorption of water vapor, and the equilibrium adsorption capacity is significantly reduced at low concentrations of air pollutants despite the high surface area of the adsorbents. Other technologies, such as ultraviolet radiation, ionization, and nonthermal plasma decomposition, may generate ozone as a harmful by-product. Thermal-catalytic degradation is effective but energy intensive. Biodegradation usually requires large-scale facilities, and its activity is strongly limited by environmental factors. Considering the above limitations, photocatalytic oxidation (PCO) is proposed as an ideal technology for air purification because it can degrade diverse air pollutants into non-toxic or less harmful forms using solar (or artificial) light under ambient conditions^2^. The photocatalyst (PC) process (eq 1) has some intrinsic similarity to the self-cleaning mechanism in Earth’s atmosphere (eq 2) in that both are based on indirect (sensitized) photooxidation to generate in situ oxidants (e.g., •OH) in air.

1 $${\mathrm{PC}}\,({\mathrm{heterogeneous}}\,{\mathrm{sensitizer}}\,{\mathrm{such}}\,{\mathrm{as}}\,{\mathrm{Ti}}{{\mathrm{O}}}_{2})+{{\rm{H}}}_{2}{\rm{O}}(g)+h\nu\,\,{\to }\,\,{\scriptstyle{\bullet}} {\rm{OH}}$$

2 $${\rm{O}}_{3}({{\mathrm{molecular}}\,{\mathrm{sensitizer}}})+{{\rm{H}}}_{2}{\rm{O}}(g)+h\nu\to {2}\,{\scriptstyle{\bullet} }{\rm{OH}}+{{\rm{O}}}_{2}$$

The prominent advantages of photocatalytic air purification are (1) no need for chemicals or external energy input except light, which is not costly when utilizing ambient light or sunlight, (2) safe operation under ambient conditions and relatively humidity-insensitive activity, and (3) the ability to fully mineralize volatile organic compounds (VOCs) to CO_2_ and H_2_O. On the other hand, this process suffers from low photon utilization efficiency and slow removal rate, difficulty of scaling up, and fouling/deactivation of photocatalysts during prolonged operation.

## Materials and process characteristics in photocatalysis

The best feature of photocatalysis, which distinguishes it from thermal catalysis, is the need for photons, the flux of which limits the overall process. As a result, many PCO reactions are more limited by the photon flux than by the active surface area, unlike most heterogeneous catalysis methods. Photocatalysts absorb photons to generate pairs of electrons and holes that react with dioxygen, water, and surface hydroxyl groups to generate reactive oxygen species (ROS), such as •OH, O_2_•^−^/HO_2_•, ^1^O_2_, and H_2_O_2_, which are the key oxidants that decompose air pollutants. As the majority of electron-hole pairs are rapidly recombined, only a tiny fraction of them (less than 1% in many cases) successfully induce PCO reactions. Much effort has been made to improve the photon utilization efficiency for decades, but the successful performance is still limited. The most studied approach is to extend the photocatalyst’s light absorption edge into the visible light range so that more photons can be used^3^. Analysis of the research literature (published in 1999–2018) on air-purifying photocatalysts shows that modified TiO_2_ accounts for the largest share (55.9%), followed by Bi-based materials (11.9%) and WO_3_ (7.3%), among the studied visible-light photocatalysts^2^. Regarding modified TiO_2_ materials with visible light activity, most studies have investigated impurity doping and heterojunctions with narrow-bandgap semiconductors or metal nanoparticles. These methods are also useful for increasing the charge separation efficiency and subsequently generating more ROS. It is interesting to note that TiO_2_-based photocatalysts are still the most studied and the most practical option for air purification applications despite the strong emphasis on the development of new and novel visible light-active materials in academic research. Although modified TiO_2_ is generally not a strong absorber of visible light, the strong oxidation potential of the TiO_2_ valence band (VB) edge, along with its excellent stability, low cost and low toxicity, makes it a practical photocatalyst. As a result, a majority of photocatalytic air purification application studies have employed pure and modified TiO_2_, which does not seem likely to be replaced by new photocatalytic materials in the near future^4,5^.

The clear trend in photocatalytic material development research is to search for inexpensive and abundant materials with high visible light activity as an alternative to TiO_2_ to make the technology more feasible. One popular candidate is carbon-based materials such as g-C_3_N_4_ and its derivatives, and various carbon nanomaterials such as reduced graphene oxide, carbon nanotubes, and nanodiamond have also been tested to replace costly noble metal cocatalysts (e.g., Pt, Au, Pd)^6,7^. However, carbon-based materials suffer from low photoactivity and long-term instability under irradiation due to their tendency to be photooxidized. Although more efficient visible light photocatalyst materials have been extensively tested, the redox power of excited electrons and holes in visible light photocatalysts is lower than that of UV-active photocatalysts. Using less energetic photons results in lower redox power; therefore, it should be noted that visible light photocatalysts are not always the best practical solution for air purification purposes.

**Fig. 1:**
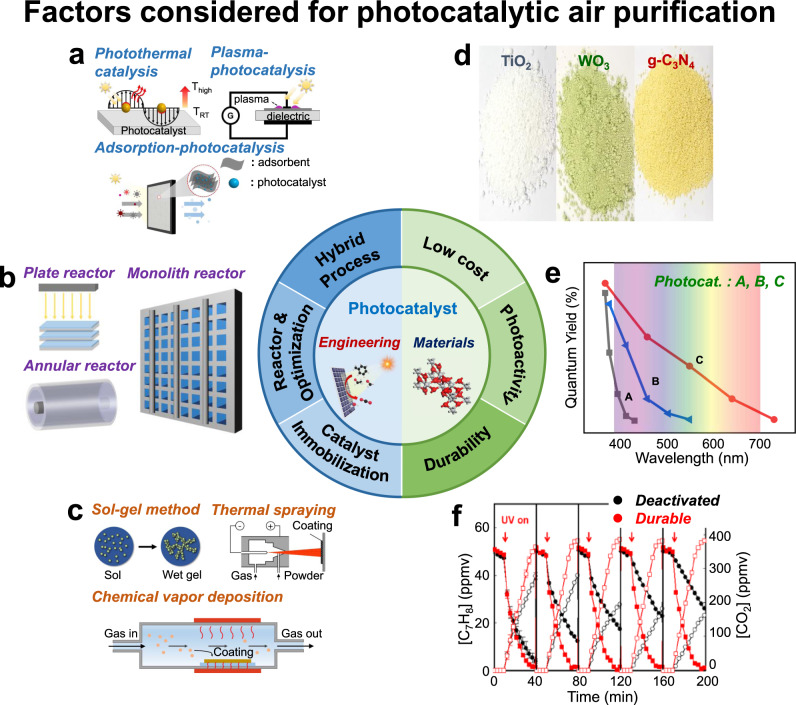
Holistic approach to the practical applications of photocatalytic air purification with considering various engineering and materials aspects. **a** hybridization of photocatalysis with other technologies, **b** photocatalytic reactor development, **c** photocatalyst immobilization methods, **d** low-cost photocatalytic materials, **e** photocatalytic activities as a function of the wavelength, **f** durability of photocatalysts against deactivation and fouling. This figure content is adapted with permission from Weon et al.^4^ Copyright (2018) American Chemical Society.

Another important issue in developing air-cleaning photocatalytic materials is the durability of the materials, which has been far less considered than the photoactivity in most studies, although it is the most critical factor for practical application. It is commonly observed that photocatalysts are gradually deactivated during photoreactions. This can be caused by the intrinsic instability of the photocatalytic material but more often by catalyst surface fouling as a result of the accumulation of recalcitrant intermediates and products. Such catalyst fouling during air treatment is more serious than in the fouling of aqueous-phase photocatalysis, where water as a solvent dissolves the degradation products and intermediates and prevents their surface accumulation. Photocatalyst surface fouling is most often observed during the degradation of aromatic VOCs and heteroatom-containing (e.g., N, S, and P) VOCs as a result of the accumulation of recalcitrant and non-volatile products^5,8^. In addition, the real-world application of photocatalytic air cleaning should take into account the presence of nuisance components such as dust and aerosol particles that rapidly foul the photocatalyst surface, which seriously limits the outdoor application of this method. Ensuring the long-term durability of photocatalysts in the real world beyond laboratory conditions presents the greatest challenge in commercializing this technology, which has received little attention thus far. The development of practical photocatalytic air purification systems should require a holistic approach that integrates material design/preparation with diverse compositions, structures, and morphology; reaction condition optimization; reactor design and engineering; and hybridization with other technologies (Fig. 1).

Photocatalytic decomposition reactions of various chemical compounds are highly substrate-specific and strongly depend on the molecular structure and composition of target compounds as well as the kind of photocatalyst^8,9^. There are a wide variety of air pollutants whose photocatalytic removal efficiencies vary greatly, and an enormous number of published studies have reported the photocatalytic degradation of air pollutants. A Google Scholar search for research articles on “*photocatalytic degradation of air pollutants*” shows 195,000 results! The main problem in analyzing these published results is that it is very difficult to directly compare photocatalytic activity data that were measured with different methods and experimental conditions. Therefore, there is a strong need to standardize the measurements and assessments to compare the photocatalytic activity data reported from different laboratories. To the best of our knowledge, the current air purification ISO methods are only available for the removal of NO, acetaldehyde, toluene, formaldehyde, and methyl mercaptan, but very few studies follow these standards^10^. More unified and standardized test methods for photocatalytic air purification have yet to be established.

## Some practical considerations for commercial applications

The design, optimization, and scale-up of photocatalytic reactors are challenging issues for the commercialization of photocatalytic air purification. Optimization of the PCO reactor is more complex than that of typical heterogeneous catalytic reactors, as the PCO process should consider both mass transfer and light delivery parameters. An ideal reactor should allow a sufficient number of photons to reach the full surface area of the photocatalyst to maximize the overall air treatment efficiency. The first essential step in preparing practical photocatalytic reactors is to immobilize a durable and robust photocatalyst layer on the support surface. Photocatalysts can be immobilized by various methods, such as the sol-gel technique, thermal spraying, chemical/physical vapor deposition, and electrophoretic deposition, which is usually followed by high temperature roasting to achieve higher crystallinity and stronger adhesion on the support^11^. This thermal treatment is technically simple but presents a significant hurdle for the practical fabrication of scaled-up reactors because it demands significant energy consumption and a large furnace or heating facilities, which should increase the overall cost. Therefore, the photocatalyst immobilization process without thermal treatment is highly desirable, but remains challenging. The successful development of an ambient-temperature immobilization process will be a breakthrough in facilitating the commercial applications of photocatalysts for various purposes.

For reactor design, plate and annular reactors are usually used in laboratory studies, but they are not suitable as practical reactors because of their low air throughput and reaction area. Monolith-type reactors with a compact structure have high throughput and a low pressure drop, but the light intensity is rapidly extinguished through the monoliths, hindering the uniform illumination of the catalyst surface. To improve the performance of the reactors, upgraded reactors such as multiplate reactors, multiannular reactors, parallel-channel monolith reactors, and radial-leakage optical fiber honeycomb reactors have been developed^12^.

Despite many advantages, photocatalytic air purification has serious limitations, such as a slow rate of treatment. The combination of photocatalysis with other technologies, such as adsorption-photocatalysis, photothermal catalysis, and plasma photocatalysis, has been proposed as a promising method to provide synergistic advantages. Hybridization of an adsorbent and a photocatalyst should increase the treatment capacity by rapidly capturing incoming target compounds on the catalyst/adsorbent surface, especially when the photocatalytic degradation capacity cannot match the rapid influx of target compounds onto the surface in real time. The immediately adsorbed target molecules can be gradually degraded on the photocatalytic active sites by regenerating the adsorbent surface^13^. The capture-and-degradation strategy can overcome the imbalance between the rapid influx and the slow photocatalytic degradation of target compounds. Photothermal catalysis combines the high efficiency and durability of thermocatalytic oxidation with the low energy consumption of photocatalytic oxidation^14^. Plasma promotes the degradation of air pollutants, and photocatalysis reduces the formation of undesired by-products (e.g., NO_*x*_ and O_3_) that are often produced in plasma-driven catalysis^15^. Despite distinctive advantages, hybrid processes are still at an early stage, and more in-depth studies are required to elucidate the synergistic mechanisms and resolve practical engineering issues.

## Outlook

Using light to clean up polluted air is an ideal technology that mimics nature’s process and has great potential to be developed as a key technology for air purification, which still needs major breakthroughs in several areas. Although current academic research is heavily focused on materials development, engineering for commercialization demands more investigation of practical issues such as preventing photocatalyst fouling/deactivation, facile and low-temperature immobilization of photocatalysts on a support, and effective and economical reactor design. The extensive search for new and novel materials to replace the classic TiO_2_ material as an air-cleaning photocatalyst has not been very successful for practical purposes, and it is expected that TiO_2_-based photocatalysts will remain the mainstay material for the time being. Overall, the most suitable application of photocatalytic air purification seems to be indoor air, where pollutants are at the sub-ppm level and interfering substances such as dust and aerosols can be controlled at a minimal level. The ideal scenario for photocatalytic indoor air cleaning is to utilize ambient room light, which requires the development of more active visible-light-responsive materials. Future research on photocatalytic air purification should address practical issues more seriously to bridge the gap between laboratory findings and real-world problems.
